# Supplementary material for: Role of Nodulation-Enhancing Rhizobacteria in the Promotion of Medicago sativa Development in Nutrient-Poor Soils
Source: Plants (Basel). 2022 Apr 26;11(9):1164. doi: 10.3390/plants11091164 (PMC9099972; doi:10.3390/plants11091164)
Supplement: Supplementary file 1 [file plants-11-01164-s001.zip › plants-1664739-supplementary.pdf]

Table S1. PGP properties showed by isolated rhizobacteria.
Selected strains appear in bold.

Strain	Phosphate solubilization	Siderophore production	IAA	N fixation	Biofilm	ACC deaminase activity
**L1**	**8**	**16**	**2.051**	**+**	**+**	**-**
**L2**	**8**	**20**	**6.522**	**+**	**-**	**1.072**
**L3**	**12**	**20**	**2.069**	**+**	**-**	**1.113**
L4	14	26	2.144	-	-	1.029
L5	12	-	2.010	+	-	-
L6	8	20	2.096	+	-	1.257
L7	6	16	2.100	+	-	-
L8	14	22	2.121	+	-	2.087
L10	12	22	6.759	+	-	1.493
L11	8	20	2.091	+	+	-
L12	6	18	2.152	+	-	-
L13	6	16	2.071	-	-	-
L14	6	20	2.080	+	-	1.684
L15	18	-	11.133	+	-	-

+, presence of the property; -, absence of the property. Values of phosphate solubilization and siderophores production express the diameter of the halo in mm. Values of IAA production are expressed in $mg \cdot l^{-1}$. Values of ACC deaminase activity are expressed in μmoles α-ketobutyrate·mg $protein^{-1} \cdot h^{-1}$.

Table S2. Morphological characteristics of the isolates.
Selected strains appear in bold.

Morphological characteristics	Strain
Rod-shape Gram-negative	**L1**, **L2**, L4, L6, L7, L8, L10, L11, L12, L13, L14, and L15
Rod-shape Gram-positive	**L3**
Rod-shape and sporulated Gram-positive	L5

Table S3. Enzymatic activities detected in isolated bacteria.
Selected strains appear in bold.

Strain	DNAse	Amylase	Cellulase	Lipase	Pectinase	Protease	Chitinase
**L1**	**-**	**-**	**-**	**-**	**+**	**+**	**-**
**L2**	**-**	**-**	**-**	**-**	**+**	**+**	**-**
**L3**	**-**	**-**	**+**	**-**	**-**	**+**	**+**
L4	-	-	-	-	-	-	-
L5	-	-	-	-	-	-	-
L7	-	-	-	-	-	-	-
L6	-	-	-	-	-	-	-
L7	-	-	-	-	-	-	-
L8	-	-	-	-	-	-	-
L10	-	-	-	-	-	-	-
L11	-	-	-	-	-	-	-
L12	-	-	-	-	-	-	-
L13	-	-	-	-	-	-	-
L14	-	-	-	-	-	-	-
L15	-	-	-	-	-	-	-

+, presence of the activity; -, absence of the activity.

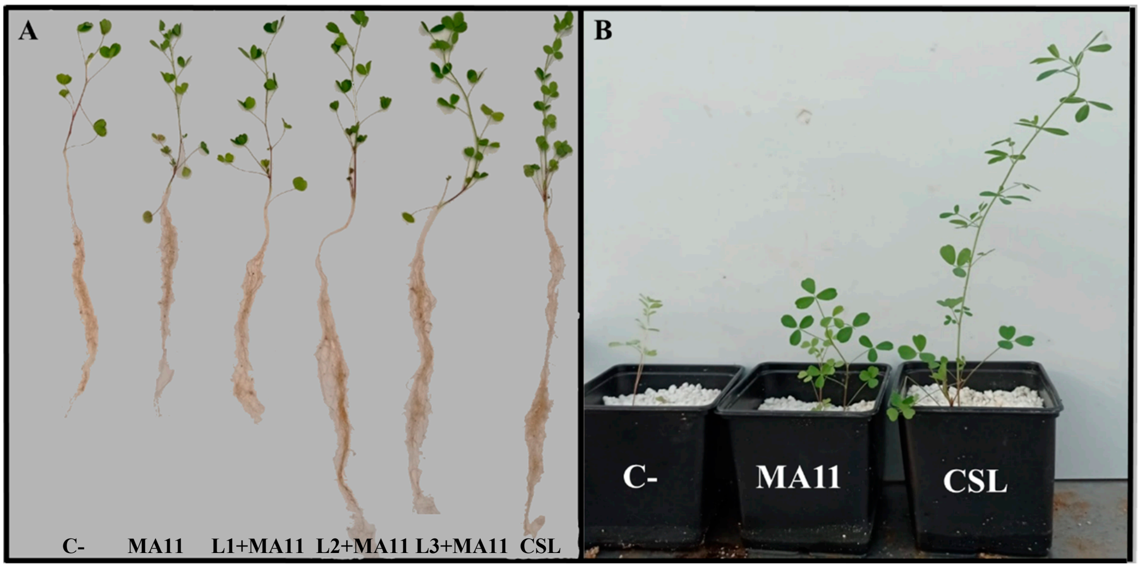

Figure S1. Comparation of plant size of the different inoculation treatments after 60 days from germination under greenhouse conditions and nutrient-poor soils as substrate. (A) Length of aerial part and roots of *M. sativa* of all the treatments; **(B)** Comparation of control plants, inoculated plants with MA11, and inoculated plants with the *consortium.* C-: without inoculation; MA11: inoculation with *E. medicae* MA11; L1+MA11: inoculation with strains L1 and MA11; L2+MA1: inoculation with strains L2 and MA11; L3+MA11: inoculation with strains L3 and MA11; CSL: inoculation with a *consortium* of strains L1, L2, L3, and MA11.

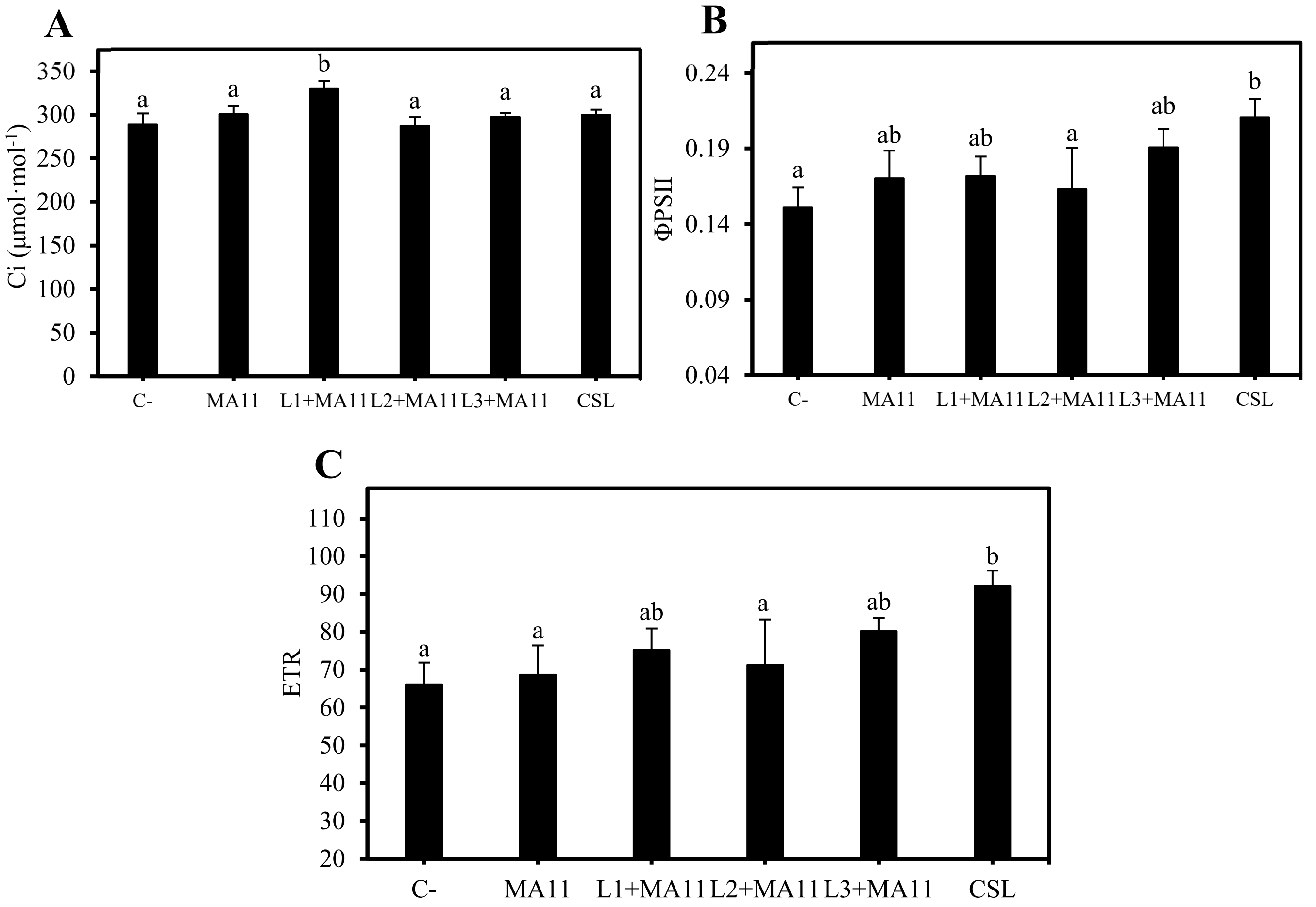

Figure S2. Photosynthetic parameters. (A) Intercellular CO_2 concentration, (B) PSII operational and maximum quantum yield, and (C) electron transport rate in plants of *M. sativa* 60 days under greenhouse conditions from germination with a nutrient-poor soil as substrate and different inoculation treatments. Values are means ± S.D. (n = 16). Different letters indicate means that are significantly different from each other (one-way ANOVA; LSD test, $P < 0.005$). C-: without inoculation; MA11: inoculation with *E. medicae* MA11; L1+MA11: inoculation with strains L1 and MA11; L2+MA1: inoculation with strains L2 and MA11; L3+MA11: inoculation with strains L3 and MA11; CSL: inoculation with a *consortium* of strains L1, L2, L3, and MA11.
